# Functional connectivity of sensory and executive function networks during a story listening task is related to parent/child interaction during joint reading: a functional MRI diffusion map study

**DOI:** 10.1007/s11682-025-01037-2

**Published:** 2025-07-02

**Authors:** Tzipi Horowitz Kraus, Marwan Bebar, Adi Jacobson, John Hutton

**Affiliations:** 1 Educational Neuroimaging Group, Faculty of Education in Science and Technology, Technion- Israel Institute of Technology, Haifa, Israel; 2 Faculty of Biomedical Engineering, Technion- Israel Institute of Technology, Haifa, Israel; 3 Kennedy Krieger Institute, Baltimore, MD USA; 4 Department of Psychology and Behavioral Sciences, Johns Hopkins University School of Medicine, Baltimore, MD USA; 5 Johns Hopkins University School of Medicine, Baltimore, MD USA; 6 University of Texas Southwestern Medical Center, Dallas Texas, USA

**Keywords:** Parent-child interaction, Executive functions, Audiovisual integration, Storytelling, Diffusion maps, Maternal depression, Joint attention, Reading

## Abstract

The quality of parent–child interaction during shared reading (“shared reading quality”) is strongly linked to cognitive and relational benefits. However, the relationship between shared reading quality and activation and synchronization of reading-related brain networks has not yet been characterized. The current study involved 22 4-year-old girls who completed functional MRI including a validated stories listening task, and a primary parent. Prior to MRI, video observation of the parent and child reading together was conducted and later coded using a standardized scoring form quantifying parent–child verbal and nonverbal interaction. Behavioral measures included demographics and a maternal depression scale. To achieve this goal, fMRI stories-listening data was utilized to create a diffusion maps algorithm and then to classify the level of parent–child interaction during the shared reading observation. The algorithm clustered children with higher parent–child engagement scores with fMRI diffusion patterns in regions of the brain known to support reading. This study establishes proof-of-concept that applying this diffusion maps algorithm to brain functional connectivity data can reliably predict parent–child interaction during shared book reading. It also suggests that an algorithmic approach may be a novel, data-driven means to quantify parent–child interaction in different contexts (e.g., reading, play) and populations.

**Supplementary Information:**

The online version contains supplementary material available at 10.1007/s11682-025-01037-2.

## Introduction

Parent–child interaction is the basis for intact cognitive and emotional child development as well as academic success, such as reading development. This interaction during book reading was found to engage neural circuits supporting language development and learning. Less beneficial interaction, such as in the case of maternal depression, can negatively affect the development of the above abilities. Using a unique dimensional reduction method, the current study aimed to determine if the degree of parent–child interaction during shared book reading could be predicted using child-brain functional connectivity data related to reading development obtained during a story listening fMRI task.

### Neurobiological correlates for stories listening

Reading, the ability to identify words and understand their meaning, is a vital skill for academic, vocational and relational success. However, since it is a relatively new skill on an evolutionary scale, there is no hardwired brain network to support reading development. Instead, brain areas that evolved to support more basic functions (e.g., language, vision) must be adequately developed and then integrated to form a “functional” reading network, which is most efficiently done during early childhood when the brain is highly plastic (Hutton et al., 2017a). On a behavioral level, this integration process is termed “emergent literacy,” a developmental continuum that begins in infancy and extends through school age (Dosenbach et al., 2008). Brain regions known to support emergent literacy and reading have been well described and include the superior temporal gyrus and the angular gyrus (linguistic abilities) (Vannest et al., 2009), frontal regions (executive functions, comprehension) (Dosenbach et al., 2008; Schmithorst et al., 2006), and visual areas including the fusiform gyrus (letter and word recognition) (Berl et al., 2010).

Interestingly, the same neural circuits supporting reading abilities are also involved in pre-reading activities, notably while listening to stories (Horowitz-Kraus et al., 2024). More nurturing home literacy environment (resources and routines supporting reading at home) has been linked to stronger activation of many of these areas during stories listening in preschool-age children using magnetic resonance imaging (MRI), such as those supporting visual processing and executive functions during (Hutton et al., 2015). Engagement of these areas has been linked to greater imagination and comprehension abilities, and likely helps shape the future reading network (Horowitz-Kraus et al., 2024; Farah et al., 2020a). One key aspect of stories listening that has been shown to confer cognitive and relational benefits (e.g., bonding) and also linked to greater engagement of brain areas supporting language, executive function and comprehension in young children, is the level of verbal and nonverbal interaction (“shared reading quality”) (Hutton et al., 2017a). Altogether, these MRI-based studies suggest that listening to stories, especially in an interactive way, supports emergent literacy and subsequent reading skills by stimulating and enhancing functional connectivity between reading-related brain regions and networks at this formative age (Horowitz-Kraus et al., 2024).

### Stories listening: an important facilitator for parent–child interaction

Brain development is affected by a child’s interactions with their environment beginning in infancy (Fox et al., 2010). Although children have many types of environmental interactions at young ages, the most meaningful is with their parents, described as their “first and most important teachers” (Hutton et al., 2017a). Extensive behavioral and neurobiological evidence has found that shared book reading, especially involving a child and their parent, is highly beneficial for cognitive, relational and brain development, in turn supporting reading abilities and learning (Hutton et al., 2015). It has been shown that high levels of parent–child interaction during “shared” reading may be especially impactful on each of these levels, amplifying benefits and subsequent outcomes (Hutton et al., 2017a). However, there is considerable variability in the quality of parent–child reading, fueled by cultural and socioeconomic factors.

An evidence-based construct for interactive reading has been termed “dialogic” reading, which involves specific types of prompts and responses during a story (Farah et al., 2019). Higher levels of interactivity (“dialogic-ness”) has been linked to improved language, relational and cognitive abilities in children (Hutton et al., 2017a). This has been recently supported by MRI studies showing associations between interactivity during parent–child “shared” reading and engagement of neural circuits supporting language and executive functions (Hutton et al., 2017a). Other studies examining the effect of dialogic, interactive reading with children at that age, echoed these findings using EEG measures (Twait et al., 2019).

### Maternal depression and storytelling

Maternal depression is characterized by low mood, loss of interest and reduced activity (Ammerman et al., 2012). Germane to the current study, it has been shown that mothers with maternal depression tend to read less to their children (Kavanaugh et al., 2006), and when they do, tend to read less interactively (e.g., fewer facial expressions and range of tones inviting a child's attention) (Farah et al., 2020a). Maternal depression is linked to a range of adverse parenting and child health outcomes, including lower linguistic abilities (Farah et al., 2020a). Relationships between maternal depression and neurobiological measures have also been described, including lower functional connectivity in brain regions related to executive functions and visual processing during stories listening in their 4 year-old children (Farah et al., 2020a). This was attributed to less interactivity during storytelling and consequently lower engagement of functional connections between brain regions in the child that are needed to support imagination and comprehension. However, despite these data, it has not yet been determined whether children of depressed mothers have differences in brain network organization related to emergent literacy skills and future reading readiness, which is a secondary aim of the current study. One approach that can be used to test this is to define clustering characteristics of children with and without depressed mothers during stories listening using MRI diffusion maps.

### Diffusion maps as a method of data clustering

Diffusion maps (DM) are a method for finding meaningful geometric descriptions of data sets based on diffusion properties (Coifman & Lafon, 2006). This method changes the representation of data sets with many variables into a low-dimensional format to enable a classification of the data sets in a more clear and representative manner (Lederman & Talmon, 2018). This method is based on the use of eigenfunctions of Markov matrices that can construct sets of coordinates called diffusion maps in which the data can be represented in fewer dimensions. Here, if two data points on the diffusion map are closer, the relationship between those points is stronger. Moreover, the relationship between sets of data can be represented as a geometric structure derived from diffusion map coordinates (Lederman & Talmon, 2018).

The diffusion maps algorithmic approach is particularly effective for small, high-dimensional datasets due to its ability to capture intrinsic geometric properties with lower dimensionality (i.e., it is a dimensionality-reduction technique) (Coifman & Lafon, 2004). This method preserves local relationships and structures, making it robust even with limited sample sizes, as will be presented in the current study (Coifman & Lafon, 2004; Talmon et al., 2013). It has demonstrated utility in various applications, such as image processing (e.g., facial recognition) and state estimation of high-dimensional systems, including medicine and neuroscience (Shnitzer et al., 2020; Talmon et al., 2013).

The goal of the current study was to determine whether the degree of parent–child interaction during a video-observed shared book reading session could be predicted using child-brain functional connectivity data obtained during a story listening fMRI task. Analyses were focused on networks known to be involved with narrative processing to achieve higher statistical power. The primary aim was to quantify the euclidean distance differences between the brain functional connectivity matrices for maternal-child dyads with high vs low levels of verbal and nonverbal interaction. As maternal depression has been linked to lower maternal-child interactivity including during book sharing (Farmer & Lee, 2011), a secondary aim was to determine if high vs low levels of interaction during the observed book reading session predicted by maternal depression levels.

We hypothesized that: 1) applying the DM method to connectivity data from the stories-listening fMRI task would successfully cluster functional networks related to reading EL and reading abilities by maternal-child interaction (high vs. low), 2) dyads with more interactive reading and also lower maternal depression levels would show greater functional connectivity within and between reading-related networks clustered by parent–child interaction level.

## Methods

### Participants

The study involved 22 mother–child dyads, recruited from a longitudinal home injury prevention trial serving mothers from socioeconomically disadvantaged backgrounds (Cincinnati Home Injury Prevention (CHIP) trial (Farah et al., 2021)). Participating children were girls between 3.0 and 4.5 years (mean age: 4.1 years, SD = 0.2) who met the following criteria: native English speakers from monolingual households, right-handed, full-term gestation, no history of head trauma with loss of consciousness or stimulant use, and no standard contraindications to MRI. All parents provided written informed consents. The behavioral session lasted approximately 1 h, and the neuroimaging scan took 30 min to complete. The study was approved by the Cincinnati Children’s Institutional Review Board (IRB) in accordance with the Declaration of Helsinki.

### Neuroimaging protocol

MRI scans were acquired in a dedicated MRI research area at Cincinnati Children’s Hospital Medical Center, Ohio using a 3 T Phillips Achieva MRI system. Children’s preparation prior to the scan involved an explanation video and sharing a CD with the scanner’s sounds to allow a desensitization for the sounds. The research coordinator also instructed the parents to practice the procedure of lying down inside the scanner and pretending to “stay still like a statue”. The scan usually occurred until the afternoon hours to ensure the child’s cooperation during the scan. On the day of the scan, the participant practiced the scan inside a mock scanner before entering the scanner (see (Kraus & Horowitz-Kraus, 2022)).All children were awake and non-sedated during the scan.

For fMRI, a time series of 165 blood-oxygen-level-dependent (BOLD) weighted scans covering the entire brain with 38 slices in the axial plane were continuously acquired with voxel size 3.75 × 3.75x5 mm at 2-s intervals (TR = 2) during the story listening task. In addition, a 3D anatomical (T1) brain image was acquired for the co-registration to the functional scans. The T1-weighted three-dimensional anatomical scan included an inversion recovery-prepared turbo gradient-echo acquisition protocol with a spatial resolution of 1 × 1 × 1 mm^3^.

### MRI stories-listening task

The stories-listening task utilized in the current study has been used extensively by the investigators here (Horowitz-Kraus et al., 2024; Holland et al., 2010; Horowitz-Kraus et al., 2013, 2015; Hutton et al., 2017a, b).It involves 11 alternating blocks of a control and active conditions (6 and 5 each, respectively), with a duration of 30 s for each block. The active condition involves listening to short, unrhymed stories presented by a female voice and included 9,10 or 11 sentences with varying syntactic structures and vocabulary at a preschool level (see (Hutton, et al., 2017b) for the stories transcript and description). For the control condition, a backward speech condition was used with voices with a range of frequencies of 200–400 Hz. These were presented via headphones in the MRI scanner, with no visual stimulus other than a fixation cross.

### Shared reading observation

Video observations of mother–child reading were conducted in a private room adjacent to the MRI suite following the MRI scan. These adhered to a standardized protocol that included how the room was arranged (e.g., placement of a video camera, chairs and reading materials) and a script for the research coordinator. The mother and child were encouraged to relax while discharge procedures were completed, estimated to take 15 min. Mothers were informed during the consent process that video recording would be done in the room for research purposes, though not explicitly for assessment of book reading. A high-definition webcam was discreetly mounted and recorded the interactions using Microsoft Movie Maker software. The room was furnished with two comfortable chairs and a table arranged with popular magazines, a table tent displaying the Wi-Fi password, and a new children’s picture book ("The Little Engine That Could"by Watty Piper and Loren Long). If neither the mother nor the child selected the book within two minutes, the research coordinator entered the room briefly to inform them that the book was theirs to keep and to read it if they like, with no further guidance. The purpose was to see first whether the mother and child chose to read together without any prompt (or, alternatively, logged into WiFi with their mobile device), suggesting higher familiarity with and interest in reading together. The session lasted for 15 min, after which the research coordinator returned to conclude the visit.

### Behavioral measurements

Two methods were used to quantify the level of parent–child interaction during the observed book reading. The first involved a scoring template developed for teacher training in dialogic (interactive) storybook reading with young children (CONNECT Module—UNC Chapel Hill Frank Porter Graham Child Development Institute). Here, observer(s) tabulate interactive reading behaviors aligned with the evidence-based, “dialogic” reading approach, such as asking questions, inviting the child to turn pages, responding to what the child says and making fun story noises. This results in a raw score (0 to an open-ended maximum tally), with higher reflecting more interactivity. In this study, scoring of the shared reading video observations was conducted by the principal investigator and two additional scorers—a medical student and a Master’s-level research coordinator—who first completed training in dialogic reading methodology and scoring. This training included both in-person practice sessions and completion of an online module, as outlined in (Hutton et al., 2017a). Scores in practice sessions were compared until achieving a goal of over 90% inter-rater concordance. Mothers who did not read the book despite being prompted by the coordinator received a score of 0, and refusal to read was noted.

Child engagement during the shared reading task was also evaluated using a scale specifically developed by the principal investigator and reviewed by experts at Cincinnati Children’s Hospital Medical Center (Speech-Language Pathologist, pediatricians). The scoring system ranged from 0 to 3, with 0 indicating no engagement (persistent attempts to do something else), 1 reflecting limited engagement (often tries to do something else, redirected to the story easily), 2 representing substantial engagement (mostly focused on the story, occasional shifts), and 3 denoting full engagement (focused for the entire story with almost no shifts). To distinguish children with genuine interest in reading from those whose lack of engagement stemmed from maternal refusal to read, it was agreed that children exhibiting strong or sustained interest even if the mother refused would receive a minimum score of 1. However, scorers maintained discretion in assigning final scores. Practice session scoring underwent iterative refinement through critique until consensus was consistently achieved. During the scoring of actual observations, ambiguities or concerns were addressed collaboratively, and all scores were systematically double-entered into a secure REDCap® database. In addition, to determine the level of interaction challenges (per (Lederer et al., 2022)), number of times when mothers checked their phones during the shared reading task was measured, and was scored as 1 (i.e. checked the phone) or 0 (did not check the phone), respectively (see also (Hutton et al., 2017a) for the scoring method).

Maternal depression (indirect measurement): Maternal depression levels were assessed using the validated Beck Depression Inventory-II (BDI-II) (Beck et al., 1996). This questionnaire is a self-report measure for assessing the severity of depression in individuals. This self-report includes 21 items. each addressing a symptom or attitude commonly associated with depression (e.g., mood, pessimism, sense of failure, guilt, fatigue, sleep disturbance). Each has a four-point Likert scale (0–3) for symptoms in the past two weeks. The reliability and validity of the BDI questionnaire were previously reported (for example, see (Yin & Fan, 2000; Richter et al., 1998)).

### Behavioral data analysis

Independent *t*-tests were conducted for each behavioral measure and medians were calculated. Moreover, to determine the relations between parent–child interaction measures while listening to stories and the offline parent–child interaction levels, Pearson correlations between maternal depression measures and behavioral measures were conducted.

### Neuroimaging data analysis

Functional MRI data and the 3D anatomical image of each child were pre-processed using the CONN toolbox (http://www.nitrc.org/projects/conn/; (Whitfield-Gabrieli & Nieto-Castanon, 2012)). These steps included realigning and unwarping the data; slice-timing correction, co-registration between the functional image and the anatomical image, segmentation of the different tissue types in the brain, intensity normalization and spatial smoothing. Next, we applied denoising of the BOLD signal prior to the connectivity analysis. Denoising of the task-based BOLD signal was performed using the CONN toolbox prior to the functional connectivity analysis. Confounding effects were removed through linear regression of noise components, including signals from white matter and cerebrospinal fluid (extracted via anatomical CompCor), six motion parameters and their first-order derivatives, as well as outlier volumes identified using the Artifact Detection Tools (ART). Outlier volumes were defined as time points exceeding a framewise displacement threshold equal to or above 0.5 mm or a global signal Z-score above 3, in accordance with CONN’s default settings. Each outlier was modeled as a separate nuisance regressor (scrubbing) to mitigate its influence. Following confound regression, a high-pass temporal filter with a cutoff frequency of 0.008 Hz (~ 128 s) was applied to remove low-frequency drifts, while no low-pass filtering was used, consistent with best practices for task-based analyses. All denoising steps were completed before theestimation of connectivity measures.

Following those initial pre-processing stages, the functional time series image data were used to extract the activation of the different regions of interest (ROI’s) as divided by the Power’s atlas (Power et al., 2011) for calculating the changes in BOLD overtime during the stories listening task at each ROI. Participants who exhibited significant motion during their scans, resulting in volumes that couldn’t be processed with the CONN toolbox during the pre-processing step, were defined as invalid volumes. Participants with more than 80% invalid volumes were excluded (an average of 33.9% (± 27.02) frames were equal to or above the defined frame displacement and global signal thresholds and hence were removed from the analysis). Consequently, due to the high motion level of several children, only 17 participants were included in the analysis of the 22 children who participated in this study. Using the processed data of the functional MRI scans, active condition blocks of the story-listening task were used to identify the connectivity between different regions in the brain during storylistening. For each child, a connectivity matrix was created, containing the correlation of the changes in BOLD over time (during the stories listening task) between all the different ROIs as divided by the Power atlas. Since this atlas divides the brain into 264 ROIs (Power et al., 2011), the connectivity matrix of each child was created as a 2D *Consequently, due* matrix with a size of 264 × 264.

### Diffusion Maps analysis

Following a previously published method (Habouba et al., 2024), connectivity matrices either for the whole brain or for selected networks supporting storytelling (i.e. visual processing, auditory processing and executive functions networks, i.e. Cingulo-opercular and fronto-parietal, see more in the next sections) were turned into a united three-dimensional connectivity matrix with a size of 17 × 264 × 264, which served as the input for the DM algorithm. This algorithm enabled lowering the high-dimension data of each child (connectivity matrix with a size of 264 × 264) and representing it as a single data point on a set of 3D diffusion map coordinates.

For each behavioral measurement (i.e. direct assessment of parent–child interaction during storytelling and indirect measure for parent–child interaction), the median was calculated, and the scores were divided into two groups – above the median and under/equal to the median. Then, each data point was colored according to values greater than or lower than the median values of a chosen behavioral measurement. For each of the two groups, the centroid on the DM coordinates was calculated. Then, the distance between the two centroids was calculated and was mathematically characterized to allow a separation of the group by the behavioral measurement.

To determine whether the DM better cluster the groups based on whole-brain ROIs or based on the selected networks associated with storytelling, both approaches were tested, including a whole brain analysis and network-based analysis with the following combinations: 1) visual, auditory, and EF networks (cingulo-opercular, fronto-parietal); 2) visual, and EF networks (cingulo-opercular, fronto-parietal); and 3) auditory, and EF networks (cingulo-opercular, fronto-parietal). These networks were previously uses in our studies(Appel et al., 2024; Habouba et al., 2024), and the ROIs were defined in Power et al. (2011).

### Correlations between the distance of the points from the centroids and behavioral measurements

After applying the between-networks connectivity information to the DM algorithm, the distance between the centroids of each group (above the median or less/equal than the median) was calculated applying each behavioral measure (parent–child interaction during storytelling, maternal depression).

## Results

### Behavioral measures

Demographic characteristics for the study’s sample are described in Table 1.

**Table 1:** Demographic characteristics

		N	%
Gender	Female	17	100
Annual household income ($)	Under 5,000	7	41
	5,000–10,000	4	23
	10,000–30,000	3	18
	30,000–50,000	3	18
Maternal Education level	High school graduate or less	8	47
	Some college	8	47
	College graduate	1	6

### Maternal-child reading quality

Of the 17 children who participated in both the MRI session and in shared reading video observation with their mothers, eight had child engagement scores less than the median (1.67), one received a score that was equivalent to the median, and nine received a score under/equal to the median score, whereas eight had greater scores for their engagement in the shared reading task. Regarding maternal distraction, nine mothers checked their phones during the video session, and eight mothers did not. See Table 2 for a summary of scoring results.

**Table 2:** Behavioral measures from the shared reading video observation

	Mean (SD)	Median	Min–max	Number of mothers below or equal the median	Number of mothers above median
Child engagement level (direct measure)	1.57 (0.97)	1.67	0–3	9	8
Engagement challenges (direct measure)	0.47 (0.51)	0	0–1	9	8
Maternal depression; BDI (indirect measure)	12.73 (7.98)	8.67	2.67–29.27	9	8

Regarding maternal depression, of the 17 mothers who completed video observation and child MRI, eight mothers had BDI scores over the median, two had BDI scores equivalent to the median, and nine had scores under/equal to the median (median: 8.67). See Table 2.

### Correlation between parent–child interaction during storytelling measures and indirect parent–child interaction measures

A positive trend was found between maternal depression levels (BDI score) and the direct measure for parent–child interaction challenges (i.e. the number of phone checks by the mother) (r = 0.389, p = 0.06). Additionally, there was a a negative trend between maternal depression score and observed maternal-child interaction during story reading (r = −0.363, p = 0.07).

A Pearson correlation within the parent–child direct interaction measures during storytelling revealed a negative correlation between the number of phone checks and the level of child engagement (r = −0.778, p < 0.001), indicating more phone checks were related to lower interaction during storytelling. Results suggest that higher maternal depression levels are related to more phone checks and lower interaction with the child during storytelling and that more phone checks are related to lower child engagement.

### Neuroimaging results

#### Whole brain analysis

**Fig. 1:**
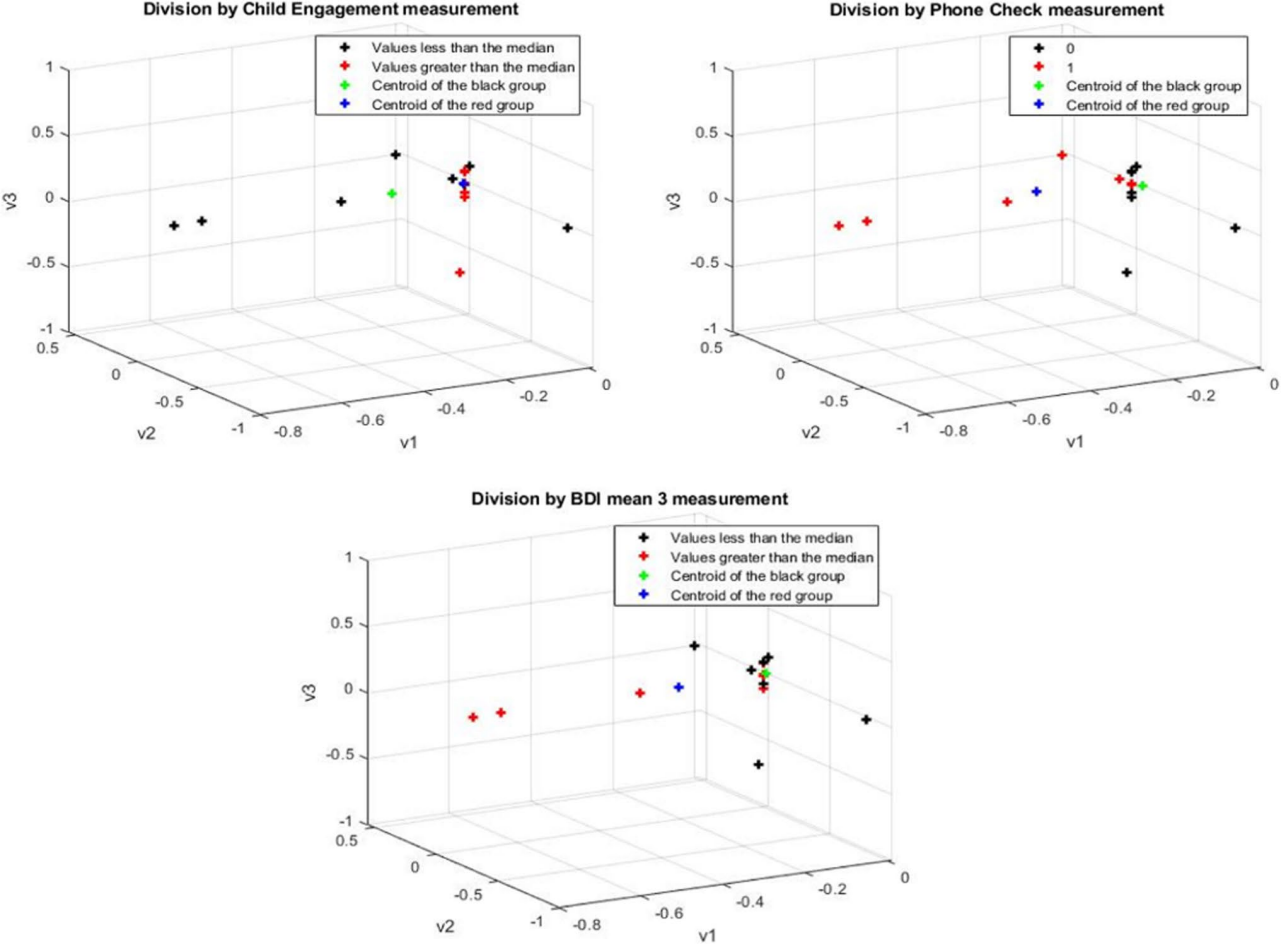
Whole brain-based diffusion maps, colored by the medians of the parent–child interaction during the direct story-listening measures (engagement with the child during storytelling and the number of phone-checks) and indirect parent–child interaction measures (maternal depression)

The whole brain matrices included in the DM algorithm, demonstrated a successful classification of the participants by the “Child Engagement” and “Phone Check” direct behavioral measurements as the input. The DM algorithm also successfully clustered the participants by their maternal depression level (BDI measurement). See Fig. 1.

Fig. 1 whole brain analysis during story listening. The black color represents datasets with values lower than the median; red color: values greater than the median. The green color represents the centroid for datasets with values lower than the median and the blue color represents centroids for datasets with values greater than the median. The upper maps represent the data for the division by parent–child interaction during storytelling (left; child engagement, right; number of times the mother checked the phone). The lower map represents the data for the division by indirect mother–child interaction (i.e. maternal depression)

### DM containing the combination of the Visual, FP, and CO networks

**Fig. 2:**
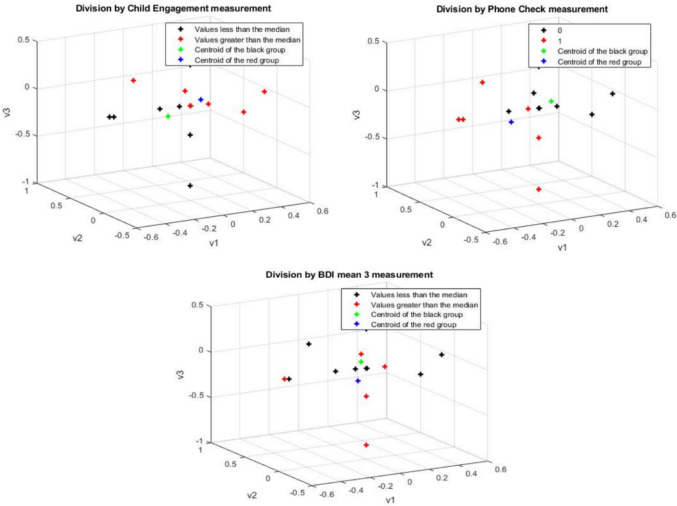
Visual processing and EF networks-based diffusion maps, colored by the medians of the parent–child interaction during direct story-listening measures (number of phone-checks, engagement with the child during storytelling) and indirect parent–child interaction measures (maternal depression)

Importing into the DM the specific functional networks for the ROIs in the selected visual processing and EF networks (FP, CO) during the stories-listening task, and coloring them based on the median values above and below the three selected measurements, the DM algorithm was able to split the full cohort into the two groups of high vs low interaction (i.e. successfully classified) the participants by the “Child Engagement” and “Phone Check” online behavioral measurements. See Fig. 2.

Fig. 2 visual processing and EF networks-based DMs. The black color represents datasets with values lower than the median; red color: values greater than the median; green color; centroid for datasets with values lower than the median and the blue color represents centroids for datasets with values greater than the median. The upper maps represent the data for the division by parent–child interaction during storytelling (left; child engagement, right; number of times the mother checked the phone). The lower map represents the data for the division by indirect mother–child interaction (i.e. maternal depression)

### DM containing the combination of the Auditory, FP, and CO networks

**Fig. 3:**
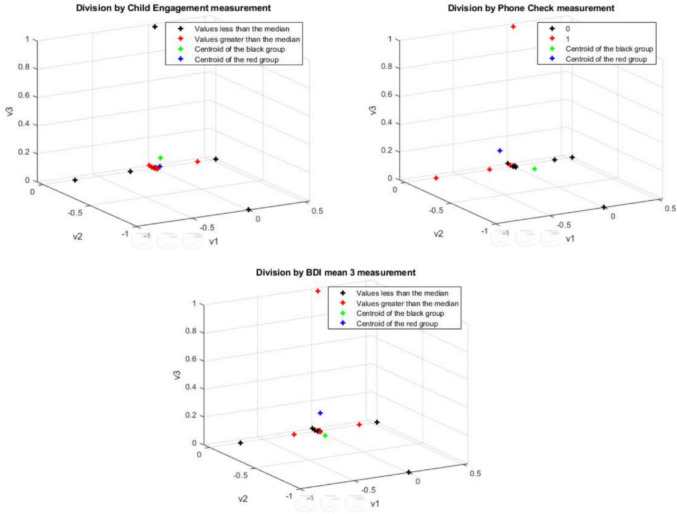
Auditory processing and EF networks-based diffusion maps, colored by the medians of the parent–child interaction during the direct story-listening measures (number of phone-checks, engagement with the child during storytelling) and indirect parent–child interaction measures (maternal depression)

Importing into the DM the functional connectivity networks from the auditory processing and EF networks (FP, CO) during the stories-listening task, and coloring them based on the median values above and below the three selected measurements, the DM algorithm successfully classified the participants only by the “Child Engagement” behavioral measurement and was not sensitive for the differences between the groups while focusing on the number of phone checks. See Fig. 3.

Fig. 3 auditory processing and EF networks-based DMs. The black color represents datasets with values lower than the median; red color: values greater than the median; green color; centroid for datasets with values lower than the median and the blue color represents centroids for datasets with values greater than the median. The upper maps represent the data for the division by parent–child interaction during storytelling (left; child engagement, right; number of times the mother checked the phone). The lower map represents the data for the division by indirect mother–child interaction (i.e. maternal depression)

### DM containing the combination of the auditory, visual, FP, and CO networks

**Fig. 4:**
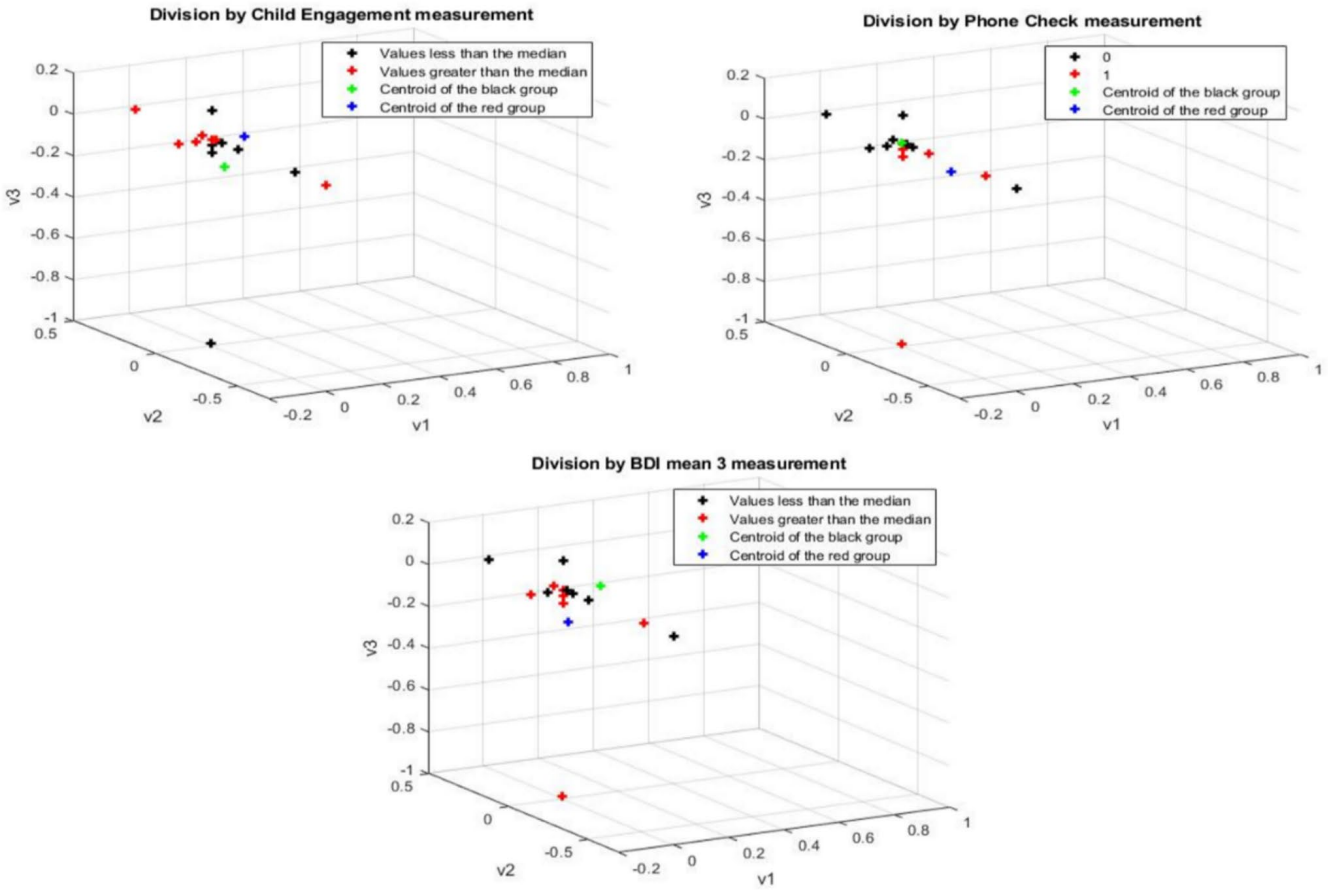
Visual, and Auditory processing and EF networks-based diffusion maps, colored by the medians of the direct parent–child interaction during story-listening measures (number of phone-checks, engagement with the child during storytelling) and indirect parent–child interaction measures (maternal depression)

Importing into the DM the functional connectivity networks from the auditory and visual processing and EF networks (FP, CO) during the stories-listening task, and coloring them based on the median values above and below the three selected measurements did not show successful clustering. See Fig. 4.

Fig. 4 visual and auditory processing and EF networks-based DMs. The black color represents datasets with values lower than the median; red color: values greater than the median; green color; centroid for datasets with values lower than the median and the blue color represents centroids for datasets with values greater than the median. The upper maps represent the data for the division by parent–child interaction during storytelling (left; child engagement, right; number of times the mother checked the phone). The lower map represents the data for the division by indirect mother–child interaction (i.e. maternal depression)

### Correlations between the distance from the centroids of each group, per DM condition and behavioral measures

Table 3 demonstrates the distances between centroids of each group (low vs high parent–child interaction), for each networks’ combination and parent–child interaction condition. The largest distance between centroids were found for the combination of the visual, CO, and FP networks, by the parent–child interaction measures during storytelling (i.e. “Child Engagement” and “Phone Check” measurements), which were 0.3067 and 0.3187, respectively. These results indicate that the DM algorithm best classified the neural matrices by these behavioral measurements, into two different clusters. The distances between the centroids, separating the neural data by the indirect parent–child interaction (BDI measurement), were nearly close, for all the between-networks analyses, which indicates that the DM algorithm could not cluster more successfully between the different between-networks analyses.

**Table 3:** Distances between the centroid

	Whole brain			Visual-EF			Auditory-EF			Visual-Auditory-EF		
	Indirect	Direct		Indirect	Direct		Indirect	Direct		Indirect	Direct	
	BDI	Child Engagement	Phone Check	BDI	Child Engagement	Phone Check	BDI	Child Engagement	Phone Check	BDI	Child Engagement	Phone Check
Distance between centroids	0.206	0.185	0.264	0.196	0.306	0.318	0.168	0.188	0.279	0.192	0.158	0.214

Table 3 the distances between centroids for each parent–child interaction measure (direct and indirect) for each networks combinations are presented in the table

## Discussion

This study aimed to determine the feasibility of DM to cluster children in terms of more vs less interactive maternal-child shared reading, based on brain connectivity patterns captured using fMRI during a stories-listening task. This approach allows an objective clustering of groups with shared characteristics, without the need to down-sample the data or reduce its dimensionality.

In line with our hypothesis, using fMRI connectivity data involving ROIs of interest, the DM algorithm successfully classified the children in terms of parent–child interaction level as measured using both “Child Engagement” and “Phone Check” behavioral measurements during the shared-reading task. The best classification (compared to a whole brain approach or other networks combinations) was obtained when importing the connectivity matrix of the visual processing and EF networks to the DM algorithm, quantified via larger distances between centroids of each group.

However, contrary to our hypothesis, the DM algorithm did not successfully classify the children by maternal depression level. We attribute this to the moderate correlation between higher maternal depression level and behavioral measures (phone check, reading engagement), which may not be sensitive enough to differentiate children of mothers with maternal depression. Additional behavioral measures might be needed for this purpose (i.e. the level of eye contact, touch, etc.).

### The importance of sensory processing and EF in stories engaging

The results of the current study are in line with previous findings highlighting the involvement of neural networks related to sensory processing and EF while listening to studies to children’s engagement in listening to the story (Farah et al., 2020b; Hutton et al., Sep 2015; Hutton et al., 2017a). These studies, as well as ours, strengthen the evidence that stories listening is not a passive process. Indeed, robust engagement in shared reading involves both imagery (i.e. visual processing) and cognitive control (i.e. EF), neural networks that are engaged via higher parental interactivity. Critical questions are raised regarding the importance of parent–child engagement during storytelling in populations that engage these networks differently than in typical populations. These include children with reading difficulties who share challenges in engaging the visual cortices (Schlaggar & McCandliss, 2007) or children with attention difficulties who share challenges in engaging neural networks related to EF (Cortese Sep, 2012). Moreover, it is unclear whether interventions such as Dialogic Reading that are intended to enhance interactivity during storytelling (Twait et al., 2019; Zevenbergen & Whitehurst, 2003) also can fuel higher engagement and connectivity in these networks. Additional studies should address this question.

### Limitations

This study has several limitations that should be noted. First, the results are based on a relatively small sample size (n = 17), limiting statistical power. Although DM successfully clustered the group with low vs high BDI scores (and found to outperform PCA methods, see supplementary materials), conducting a study with more participants would possibly improve the DM algorithm's ability to cluster the fMRI data and would likely better differentiate connectivity matrices of participants.

Additionally, the repetition time during the scans in this study was 2 s (TR = 2), which may present certain limitations. The relatively long TR reduces temporal resolution, making it challenging to capture rapid neural events or resolve closely spaced brain activities. The slower data acquisition may also extend the total data acquisition, potentially increasing the risk of motion artifacts due to the participant’s fatigue. Moreover, the fMRI sequence includes a relatively large voxel size, limited spatial resolution. Furthermore, in the current study, no strong correlation was found between the depression measure (BDI) and behavioral measures of children during the shared reading task. A future study should reassess the correlation of those measurements and determine the ability of the DM algorithm to cluster participants by this measure or others. Moreover, the main (direct) measure for child engagement involved direct observation of maternal-child interaction during storytelling, while the offline measure for engagement was the BDI questionnaire, which may not be optimally aligned. Lastly, the focus of this study was clustering participants by the DM algorithm based on several between-networks information. Although the DM algorithm successfully clustered participants here by applying the neural information of the Vis-CO-FP networks, there are additional networks that may more efficiently cluster participants, which is worthy of further study.

### Conclusions and future directions

The current study suggests that the DM algorithm has the potential to predict the quality of parent–child interaction using fMRI connectivity data. By conducting the neural information of the Visual processing, CO and FP networks, the DM algorithm successfully clustered children by two direct behavioral measures (dialogic interactivity, phone checking) during observed shared reading with their mothers. The results suggest that the DM algorithm may be able to represent visually connections between neurological and behavioral information related to shared book reading. Additional, potential applications of this data-driven algorithmic approach could include populations of interest (e.g., behavioral conditions, poverty) and other cognitive domains.

## Aclnowledgement

The authors would like to thank Prof Ronen Talmon, from the Technion- Israel Institute of Technology, for his contribution.

## Supplementary Information

Below is the link to the electronic supplementary material.

Supplementary file1 (DOCX 234 KB)

## Data Availability

Data will be available upon request from the corresponding author (THK).
