# Supplementary material for: Functional connectivity of sensory and executive function networks during a story listening task is related to parent/child interaction during joint reading: a functional MRI diffusion map study
Source: Brain Imaging Behav. 2025 Jul 2;19(6):1133–45. doi: 10.1007/s11682-025-01037-2 (PMC12831693; doi:10.1007/s11682-025-01037-2)

**Supplementary material**

*A comparison of clustering using DM vs PCA*

*Condition: Phone check (networks: visual processing, CO and FP)*

Group 0: 9 subjects

Group 1: 8 subjects

Diffusion Maps results for the Phone check condition:

Group 0: Mean distance from centroid = 0.2534 ± 0.1581

Group 1: Mean distance from centroid = 0.4074 ± 0.2296

Distance between centroids: 0.3187

PCA results for the Phone check condition:

Group 0: Mean distance from centroid = 6.3318 ± 6.5797

Group 1: Mean distance from centroid = 13.1715 ± 10.6583

Distance between centroids: 8.2970

The clustering quality comparison for the DM vs PCA suggested that the Diffusion Maps mean silhouette score was higher than for the PCA score (0.1404 vs 0.0835), suggesting that diffusion maps provide a better clustering (68.16% improvement). See supplemental figure 1:

**Supplemental Figure 1. A comparison between clustering using diffusion maps vs PCA for phone check**


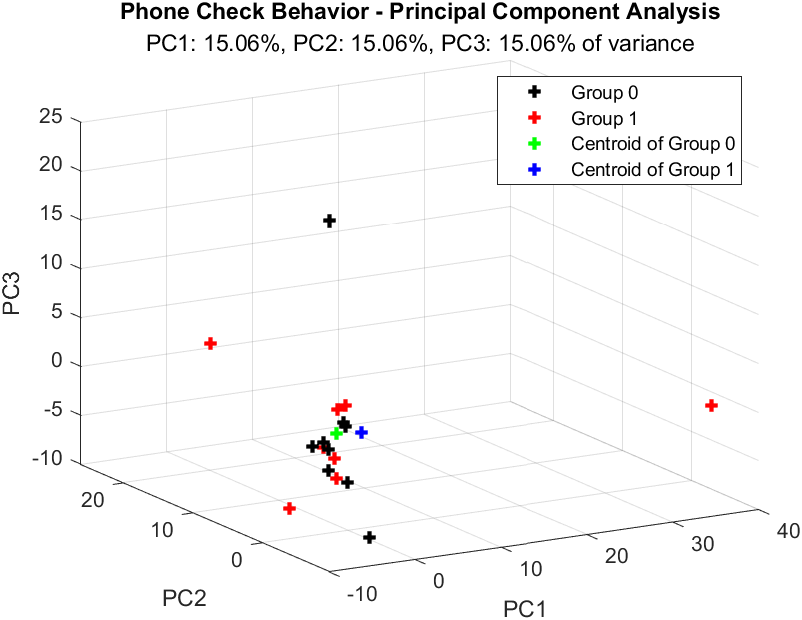

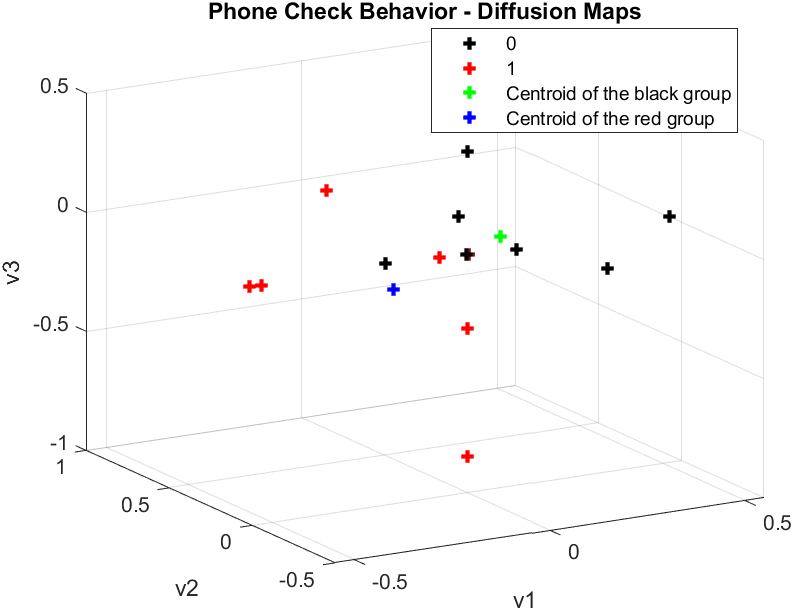


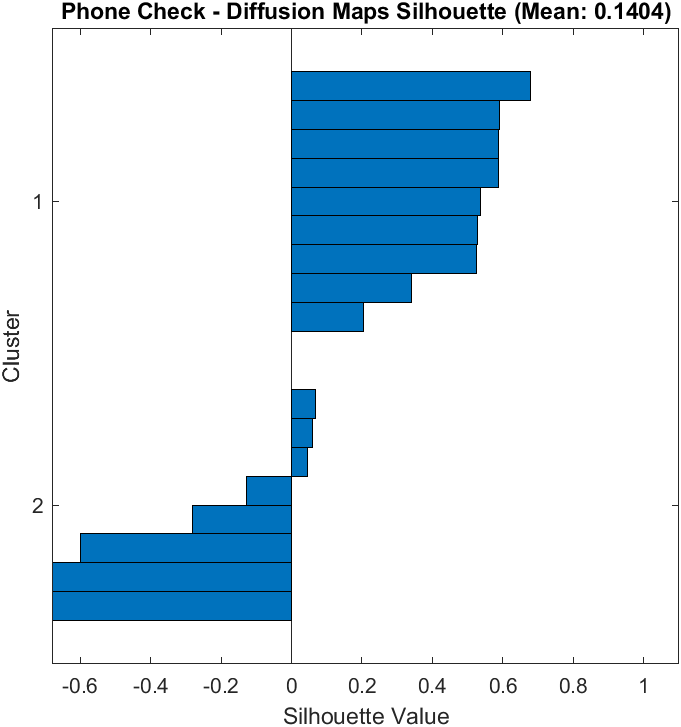

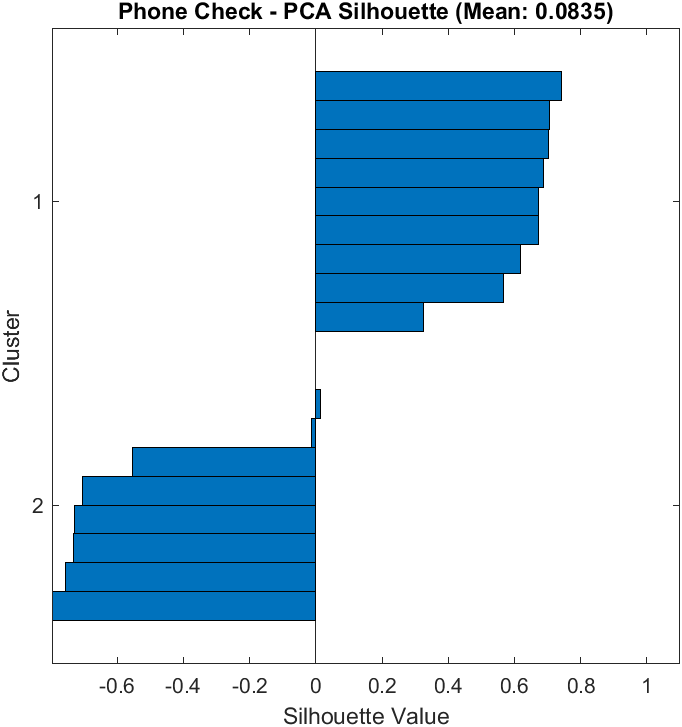


*Condition: Child engagement* *(networks: visual processing, CO and FP)*

Child Engagement median: 1.6700

Subjects below median: 9

Subjects above median: 8

Diffusion Maps results for the Child Engagement:

Group 0 (below median): Mean distance from centroid = 0.3263 ± 0.2262

Group 1 (above median): Mean distance from centroid = 0.3204 ± 0.2133

Distance between centroids: 0.3067

PCA results for the Child Engagement:

Group 0 (below median): Mean distance from centroid = 11.8445 ± 10.7431

Group 1 (above median): Mean distance from centroid = 6.6860 ± 6.6499

Distance between centroids: 8.8097

The clustering quality comparison for the DM vs PCA suggested that the Diffusion Maps mean silhouette score was higher than for the PCA score (0.0867 vs 0.0226), suggesting that diffusion maps provide a better clustering (283.35% improvement). See supplemental figure 2:

**Supplemental Figure 2. A comparison between clustering using diffusion maps vs PCA for child engagement**


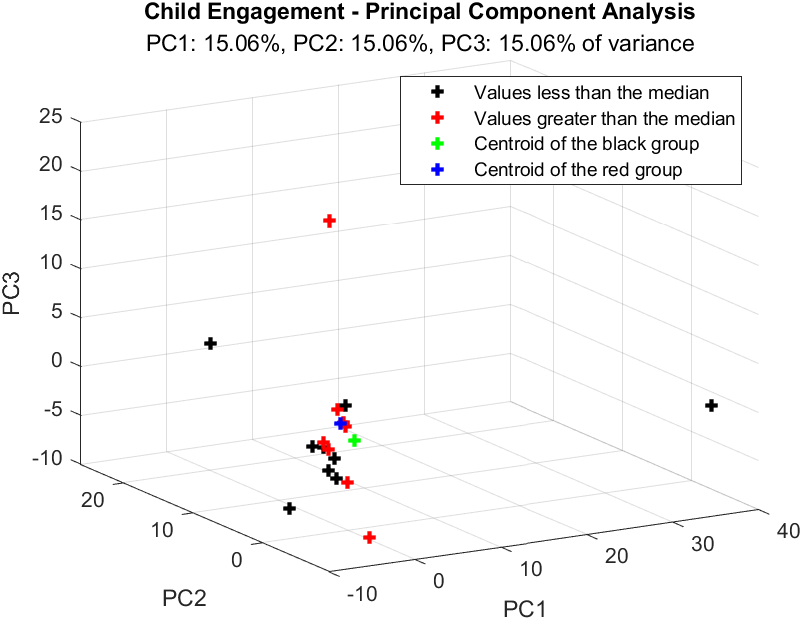


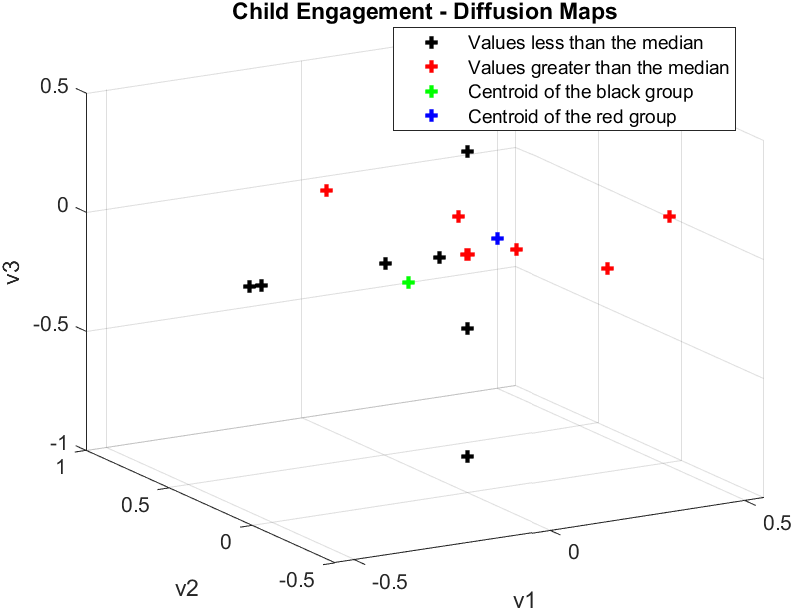


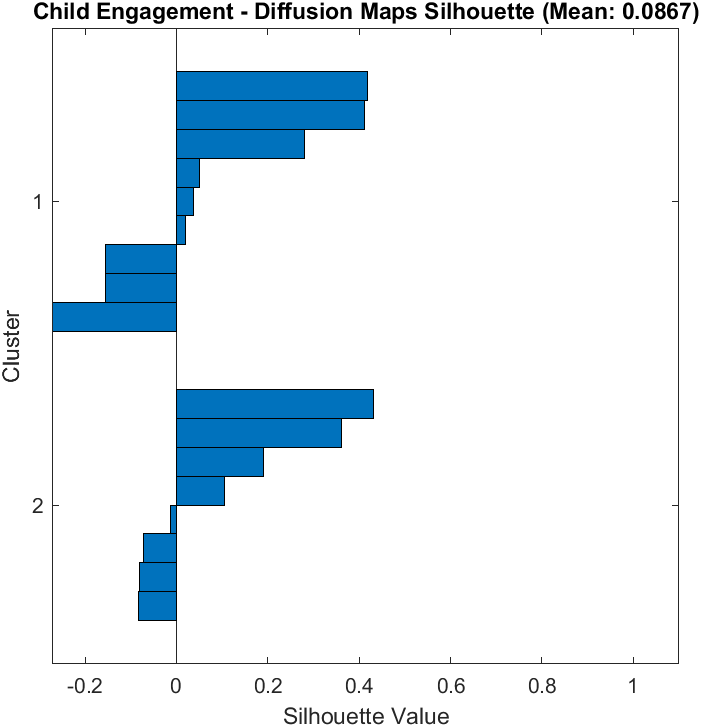

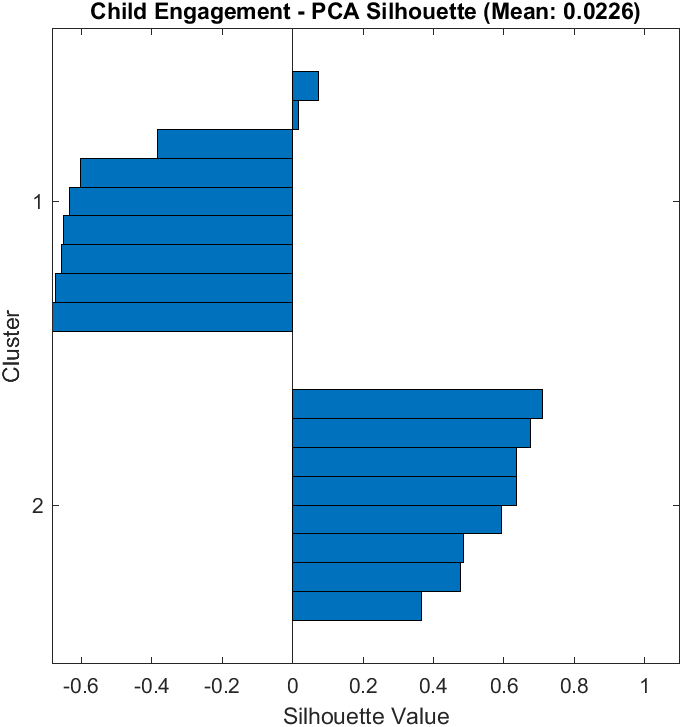

Supplement: Supplementary file 1 — Supplementary file1 (DOCX 234 KB) [file 11682_2025_1037_MOESM1_ESM.docx]
